# Therapeutic and prophylactic effects of Qipian on COPD in mice: the role of lung and gut microbiota

**DOI:** 10.1128/spectrum.01969-24

**Published:** 2025-07-14

**Authors:** Guangxia Sun, Wanming Hao, Qinghai Li, Yan Ma, Fenglan Sun, Yi Su, Xinjuan Yu, Wei Han

**Affiliations:** 1 Qingdao Municipal Hospital, Nanjing Medical University 12461, Qingdao, China; 2 Department of Respiratory and Critical Care Medicine, Qingdao Hospital, University of Health and Rehabilitation Sciences (Qingdao Municipal Hospital) 12648 , Qingdao, China; 3 Qingdao Key Laboratory of Common Diseases, Qingdao Hospital, University of Health and Rehabilitation Sciences (Qingdao Municipal Hospital) 12648 , Qingdao, China; 4 Clinical Research Center, Qingdao Hospital, University of Health and Rehabilitation Sciences (Qingdao Municipal Hospital) 12648 , Qingdao, China; Chengdu University, Chengdu, Sichuan, China

**Keywords:** chronic obstructive pulmonary disease, Staphylococcus and Neisseria tablet, gut microbiota, lung microbiota, toll-like receptor 4

## Abstract

The objective of this study was to investigate the therapeutic and prophylactic effects of the mixed extract of Staphylococcus and Neisseria tablets (Qipian) on chronic obstructive pulmonary disease (COPD) in mice and its relationship with lung and gut microbiota. C57BL/6 mice were divided into two models (prophylactic versus therapeutic administration). In the model of prophylactic administration, the mice were exposed to cigarette smoke (CS) for 16 weeks and divided into CS+Qipian group, CS+OM-85 group, and CS control group. Compared to the CS control group, mice in both CS+Qipian and CS+OM-85 groups had improved lung function, reduced pulmonary bullae and inflammatory cell infiltration, reduced collagen deposition around the bronchi, and decreased levels of inflammatory cytokines in the lung tissue. Qipian and OM-85 significantly increased the diversity of gut and lung microbiota and the abundance of Bacteroidetes, *Bacteroidales*, and *Lactobacillus*, as well as inhibited the toll-like receptor 4 (TLR4)/nuclear factor kappa-light-chain enhancer of activated B cells (NF-κB) pathway. There were no significant differences in the above parameters between the CS+OM-85 group and the CS+Qipian group. In the therapeutic administration model, the mice were exposed to CS for 24 weeks, and the results were consistent with those of the prophylactic administration model. Qipian administration has both preventive and therapeutic effects on COPD possibly by regulating the microbiota dysbiosis through the gut-lung axis, inhibiting the activation of the TLR4/NF-κB pathway, reducing inflammation, and improving lung function.

**IMPORTANCE:**

Gut and lung microbiota are associated with the development and progression of chronic obstructive pulmonary disease (COPD). Qipian treatment significantly increased the diversity of gut and lung microbiota and the abundance of Bacteroidetes, *Bacteroidales*, and *Lactobacillus*. In addition, the toll-like receptor 4/nuclear factor kappa-light-chain enhancer of activated B cells pathway mediated by the gut-lung axis may play an essential role in preventing and treating the pathogenesis of COPD, which allows for reduced inflammation and improvement of lung function.

## INTRODUCTION

Chronic obstructive pulmonary disease (COPD) is a chronic, progressive, inflammatory disease of the lungs that will lead to cor pulmonale and respiratory failure. It is estimated that there are at least 328  million patients with COPD. It kills more than 3 million people worldwide every year (1). COPD is the third leading cause of death in the world, following myocardial infarction and stroke (2). The China Pulmonary Health (CPH) study estimated that 99.9 million Chinese adults aged 20 years or older in 2015 had spirometry-defined COPD (3). COPD has emerged as a pressing public health concern that poses a global threat to our well-being (4). At present, the exact pathogenesis of COPD remains unclear, and there is still a lack of targeted therapies for COPD.

Currently, accumulating evidence has suggested that polyvalent bacterial lysates (PBLs) can alleviate COPD, asthma, respiratory infections, and other respiratory diseases through immune modulation (5–7). A meta-analysis has shown that oral administration of OM-85 (Broncho‐Vaxom) can alleviate respiratory symptoms in patients with acute exacerbations of COPD (7). Fraser and Poole (8) investigated the efficacy of PBLs including OM-85, RU41740 (Biostim), and Ismigen (Lallemand Pharma AG, Massagno, Switzerland) in preventing respiratory exacerbations in patients with COPD or chronic bronchitis and found that treatment with PBLs was associated with a slight reduction in the possibility of having exacerbations and a moderate decrease in the requirement for antibiotics. Staphylococcus and Neisseria tablet (Qipian; Qilu Pharmaceutical Co., Ltd., Jinan, China) is a polyvalent bacterial lysate of *Staphylococcus epidermidis*, *Moraxella (Neisseria) catarrhalis*, and *Bacillus subtilis* (9). Traditionally, it has been used to treat airway inflammatory diseases, including COPD and chronic bronchitis. However, its effects on COPD and the underlying mechanisms have not been thoroughly elucidated.

Maintaining microecological balance in the airway has been reported to play a crucial role in the prevention and treatment of COPD. Previous studies have shown that smokers or COPD patients exhibit significant differences in richness, evenness, and diversity of lung microecology compared with healthy subjects, and these parameters change with the severity and progression of COPD (10, 11). Notably, the gut has approximately 99% of commensal microbiota in humans. Some studies have shown that gut microorganisms can affect host metabolism, disease resistance, and development (12, 13). Recently, gut microbiota has been reported to have bidirectional communication with airway microbiota through diverse mechanisms, which is known as the gut-lung axis (12). The direct way gut microbiota impacts airway microbiota is that gut microbiota can enter the airway through physical pathways. What’s more, gut microbiota can modulate the immune system by producing metabolites and ligands, thereby influencing the immune responses and release of the inflammatory factors in the lung and indirectly regulating airway microbiota (14, 15). Therefore, regulating the gut microbiota might help maintain the stability of airway microecology via the gut-lung axis. A study has shown that oral administration of OM-85 can modify the composition of gut microbiota and subsequently impact lung microbiota (16). OM-85 exerts its therapeutic effects on COPD by creating favorable conditions within the mucosal microbiome interface for the growth of beneficial bacteria (16). Similarly, we hypothesized that Qipian might also exert its effects on COPD through the modulating gut-lung microbiota. However, the potential mechanism has not yet been elucidated.

Recently, the gut microbiota has been reported to play a role in lipopolysaccharide (LPS)-induced acute lung injury by regulating the toll-like receptor 4 (TLR4)/nuclear factor kappa-light-chain enhancer of activated B cells (NF-κB) pathway (17). TLR4 expression levels were significantly associated with both inflammation and pathogenic bacterial load in the lungs of COPD patients (18). Stimulated by pathogenic bacteria, TLR4 can activate NF-κB and other signaling pathways, thereby triggering immune-related inflammatory/anti-inflammatory factors, chemokines, and transcription synthesis (18). Whether Qipian affects the TLR4/NF-κB pathway has not yet been clarified.

The aim of this study is to use a mouse model of smoke-induced COPD to investigate the efficacy of Qipian in smoking-related COPD and its effects on gut and lung microbiota, as well as the underlying mechanisms.

## MATERIALS AND METHODS

### Animals

Animal experiments were conducted using 4-week-old C57BL/6 mice from Jinan Pengyue Laboratory Animal Breeding Ltd. (Jinan, China). Mice were housed in a room with a relative humidity of 50%–60%, a temperature of 24°C–26°C, a 12/12 hours light/dark cycle, and free access to food and water.

### Study design

We used the C57BL/6 mice (*n* = 34) to establish a mouse model of smoke-induced COPD. After 7 days of environmental adaptation, the mice were divided into two models (prophylactic versus therapeutic administration). In the model of prophylactic administration, the mice were exposed to cigarette smoke (CS) exposure for 16 weeks and divided into CS group (*n* = 6), CS+OM-85 group (*n* = 6), and CS+Qipian group (*n* = 8). In the model of therapeutic administration, the mice were exposed to CS for 24 weeks according to a previous study (19) to establish a COPD model and divided into the COPD group (*n* = 6) and the COPD+Qipian group (*n* = 8). During the model establishment, all the mice were placed in a ventilated plexiglass box and then exposed to smoke generated by burning seven cigarettes (Hong Jin Long, 1.2 mg nicotine, 15 mg tar per cigarette, Wuhan, China) that lasted for 2 hours. The smoke exposure was carried out two times a day and 6 days each week.

### Animal intervention

**Fig 1:**
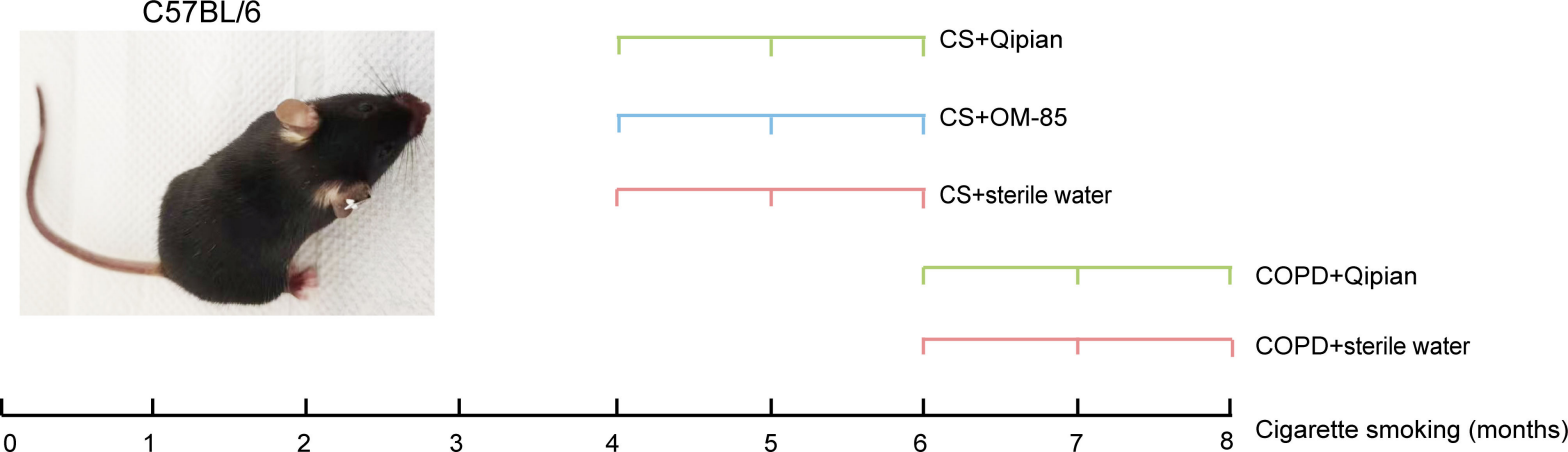
Schematic diagram of the experimental setup.

At the end of the 16 weeks of CS, the mice in the prophylactic administration model were orally administered Qipian powder (160 mg/kg), OM-85 powder (175 mg/kg), or vehicle (sterile water) once daily for 8 weeks. At the end of the 24 weeks, mice in the therapeutic administration model were orally administered Qipian (160 mg/kg) powder or vehicle (sterile water) once daily for 8 weeks. At the same time, the normal control mice were housed in clean air without any other intervention except sterile water gavage (*n* = 12) (Fig. 1).

### Lung function test

The lung function of mice in the model of prophylactic administration was performed using the Whole-body Plethysmography System (Tow-Int Tech, Shanghai, China). The mice were placed in the chamber, and their respiratory parameters during sleep were measured and recorded, including inspiratory time (Ti), expiratory time (Te), maximum inspiratory volume (PIF), maximum expiratory volume (PEF), tidal volume (TV), expiratory volume (EV), relaxation time (RT), expiratory volume per minute (MV), f (respiratory rate), forced breathing interval (PENH), and 50% expiratory flow rate (EF50). In the model of therapeutic administration, we used the EMMS Forced Maneuvers System (eSpira FM System, EMMS-CRFM100, UK) to assess the lung function of mice, with FEV25ms/FVC (forced expiratory volume at 25 ms/forced vital capacity), FEV50ms/FVC, and FEV75ms/FVC recorded.

### Lung pathology

Twenty-four hours after the final exposure, mice in both models of the prophylactic and therapeutic administration were euthanized. The lung tissues in the left lung were fixed in 10% neutral formaldehyde for 24 hours. The lung tissues were dehydrated, embedded in paraffin, and sliced into 5 µm sections on a rotary microtome. The sections were subjected to hematoxylin and eosin (HE) staining, Masson’s trichrome (MT) staining, and periodic acid-Schiff (PAS) staining (Solarbio, Peking, China). Histological changes in lung tissues were observed under a light microscope (Nikon, Japan). The obtained images were analyzed using Image J software. The morphological changes of emphysema were assessed by measuring the mean linear intercept (MLI) and mean alveolar number (MAN), as previously described (100× magnification).

### Immunohistochemistry

For immunohistochemical detection of alpha-smooth muscle actin (α-SMA) expression in the lungs, 5 µm of lung tissues was stained with anti-α-SMA (Proteintech, Wuhan, China, Rabbit polyclonal at a 1:1,000 dilution). Next, they were fluorescent-labeled with goat anti-rabbit IgG (H + L) cross-adsorbed secondary antibody (Zsbio, China). Areas that are positively immunostained for α-SMA proteins in the airway were observed under a light microscope (Nikon, Japan). The obtained images were analyzed using Image J software.

### Real-time quantitative reverse transcription PCR

Total RNA was extracted from mouse lung tissues using Trizol (Takara, Japan) reagent, and extracted RNA was subjected to reverse transcription and real-time quantitative reverse transcription PCR according to the manufacturer’s instructions (Takara, Japan). The relative value of mRNA (with glyceraldehyde-3-phosphate dehydrogenase [GAPDH] as an internal reference) was calculated by the 2^(−ΔΔCt)^ method. Primer information is provided in Table S1.

### Western blot analysis

Total proteins were extracted from mouse lung tissues, and the concentration was measured by BCA Kit (Elabscience, China). The antibodies we used were anti-NF-κB-p65 (Abcam; 1:1,000), anti-phospho-NF-κB-p65 (Abcam; 1:1,000), TLR4 (Elabscience; 1:1,000) as the primary antibodies and anti-rabbit IgG antibodies (Elabscience; 1:2,000) as the secondary antibodies. Finally, the ECL solution was applied to develop the color; a fully automated chemiluminescence gel imaging analysis system (Beijing Sage Science and Technology Co., Ltd.) was used for exposure and photography; and Image J software was utilized for image analysis.

### Microbiota analysis

After quality control, total genomic DNA was extracted from feces and the bronchoalveolar lavage fluid of mice using the TGuide S96 Magnetic Soil/Stool DNA Kit (Tiangen Biotech [Beijing] Co., Ltd.) according to the manufacturer’s instructions. The hypervariable region V3-V4 of the bacterial 16S rRNA gene was amplified with primer pairs F: 5′-ACTCCTACGGGAGGCAGCA-3′ and R: 5′-GGACTACHVGGGTWTCTAAT-3′. PCR products were checked on agarose gel and purified through Omega DNA purification (Omega Inc., Norcross, GA, USA). The purified PCR products were collected, and the paired ends (2 × 250 bp) were performed on the Illumina Novaseq 6000 platform. Firstly, Trimomatic (version 0.33) was used to perform quality filtering on the raw data, and then Cutadapt (version 1.8.3) was used to identify and remove primer sequences. The dada2 package in R was used for further quality control, including concatenation of double-ended reads and removal of chimeras. Ultimately, high-quality sequences were obtained for subsequent analysis. The sequence data reported in this study were archived in the Sequence Read Archive (SRA) database (SRA accession: PRJNA1097331, https://www.ncbi.nlm.nih.gov/sra/PRJNA1097331).

### Statistical analysis

All statistical analyses were performed with the IBM SPSS 24.0 software (IBM, New York, USA). The data were expressed as mean ± standard deviation. Student’s *t-*tests and one-way analysis of variance were used for data analysis. Multiple comparisons were performed using Tukey *post hoc* test. Correlations between variables were analyzed by the Pearson correlation test or Spearman rank correlation analysis. The Kruskal-Wallis test was employed to examine the alpha diversity at the genus level among the three groups, while the principal coordinate analysis (PCoA) was utilized to analyze the beta diversity. Differential taxonomic abundance was evaluated using linear discriminant analysis (LDA) effect size (LEfSe). The non-parametric factorial Wilcoxon rank-sum tests were utilized to perform the LEfSe analysis, with a cutoff value of *P* < 0.05 and an LDA score >2. All statistical analyses used two-sided tests, and a *P* value <0.05 was considered statistically significant.

## RESULTS

### Characteristics of the study mice

**Fig 2:**
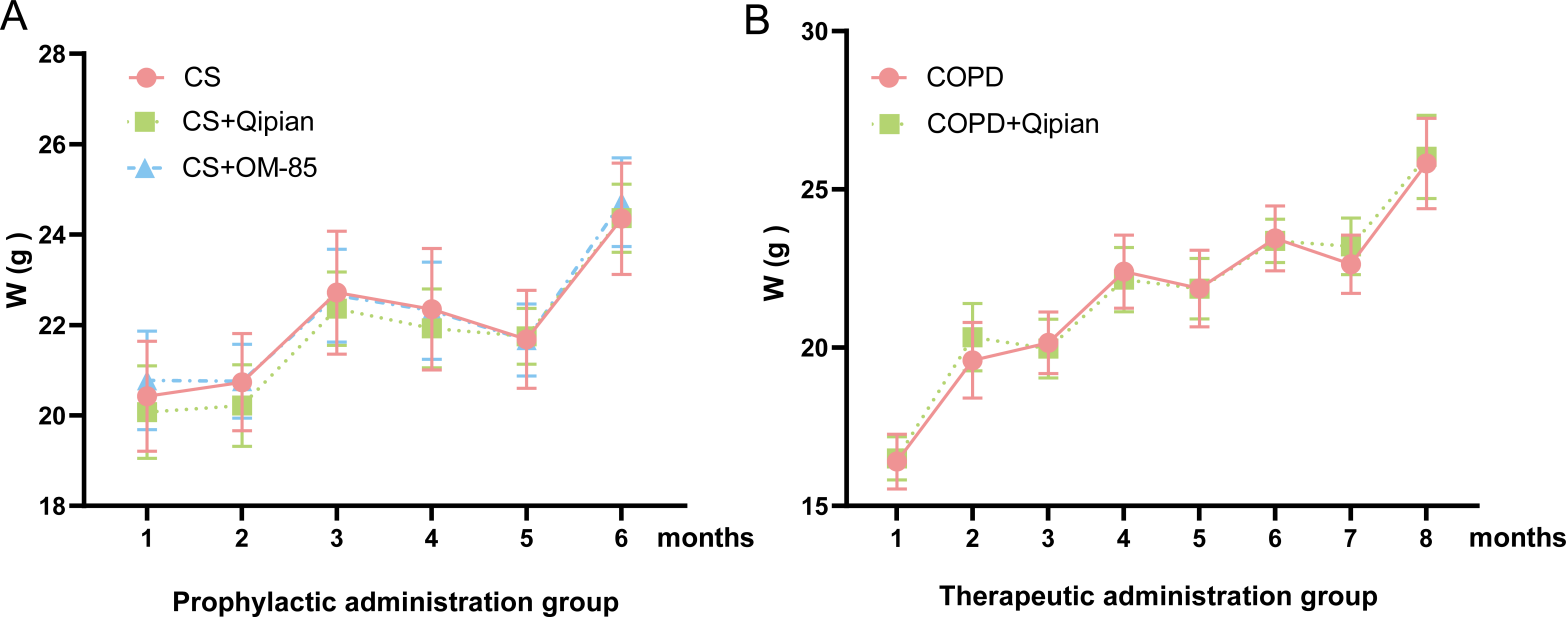
Weight changes in the two models. (**A**) Prophylactic administration model. (**B**) Therapeutic administration model.

During the experimental period, the general appearance and physical condition of mice were observed. Two months after CS exposure, abnormal symptoms such as decreased appetite, reduced food intake, shortness of breath, delayed response time, and stool abnormalities were observed in the mice. Following subsequent administration of Qipian powder and OM-85 by gavage, improvements were noted in their emotional state, fur quality, and stool consistency. Notably, compared with the CS group and COPD group, there was no significant difference in weight gain after Qipian administration (Fig. 2). Importantly, it was found that Qipian effectively alleviated shortness of breath and constipation, both of which were induced by smoking.

### Lung function

**Fig 3:**
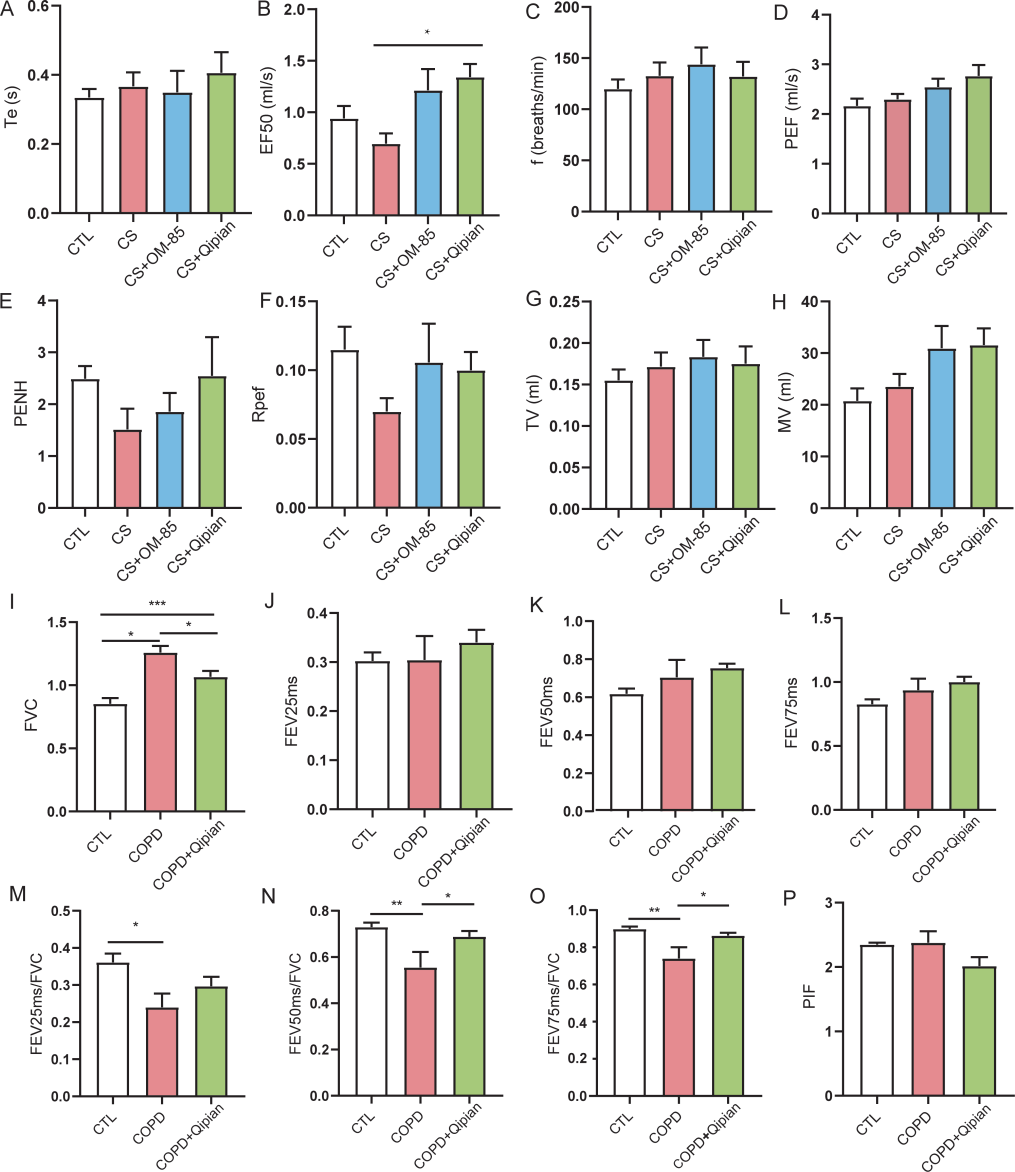
CS-induced exposure in mice resulted in alteration of respiratory parameters. (A–H) Lung function indexes of mice in the model of prophylactic administration. (I–P) Lung function indexes of mice in the model of therapeutic administration. (**A**) Te = expiration time, (**B**) EF50 = 50% expiratory flow rate, (**C**) f = respiratory rate, (**D**) PEF = maximum expiratory volume, (**E**) PENH = forced breath gap, (**F**) Rpef = time-to-peak expiratory ratio, (**G**) TV = tidal vol, (**H**) MV = expiratory vol per minute, (**I**) forced vital capacity (FVC), (**J**) forced expiratory volume at 25 ms (FEV25ms), (**K**) forced expiratory volume at 50 ms (FEV50ms), (**L**) forced expiratory volume at 75 ms (FEV75ms), (**M**) the ratio of forced expiratory volume at 25 ms to total expiratory volume (FEV25ms/FVC), (**N**) FEV50ms/FVC, (**O**) FEV75ms/FVC, and (**P**) peak inspiratory flow rate (PIF). *P < 0.05, **P < 0.01, and ***P < 0.001.

The results of lung function tests are shown in Fig. 3. After CS exposure, the EF50, PENH, and time-to-peak expiratory ratio (Rpef) of the CS group were lower than those of the control group. In the model of prophylactic administration, the EF50 of the CS+Qipian group was significantly higher than that of the CS group (*P* < 0.05) (Fig. 3B). PENH and Rpef of the CS+Qipian group were higher than those of the CS group (Fig. 3E and F). In the model of therapeutic administration, chronic CS exposure caused a significant increase in FVC (*P* < 0.001) (Fig. 3I), as well as decreases in FEV25ms/FVC, FEV50ms/FVC, and FEV75ms/FVC (all *P* < 0.05) (Fig. 3M through O), suggesting that the COPD model was successfully established. After the therapeutic administration of Qipian, FVC decreased significantly, and FEV25ms/FVC, FEV50ms/FVC, and FEV75ms/FVC increased notably (all *P* < 0.05) (Fig. 3I and M through O), indicating that Qipian could delay the changes in lung function induced by CS exposure, thereby improving lung function.

### Pathological changes and expression of inflammatory cytokines in lung tissues

**Fig 4:**
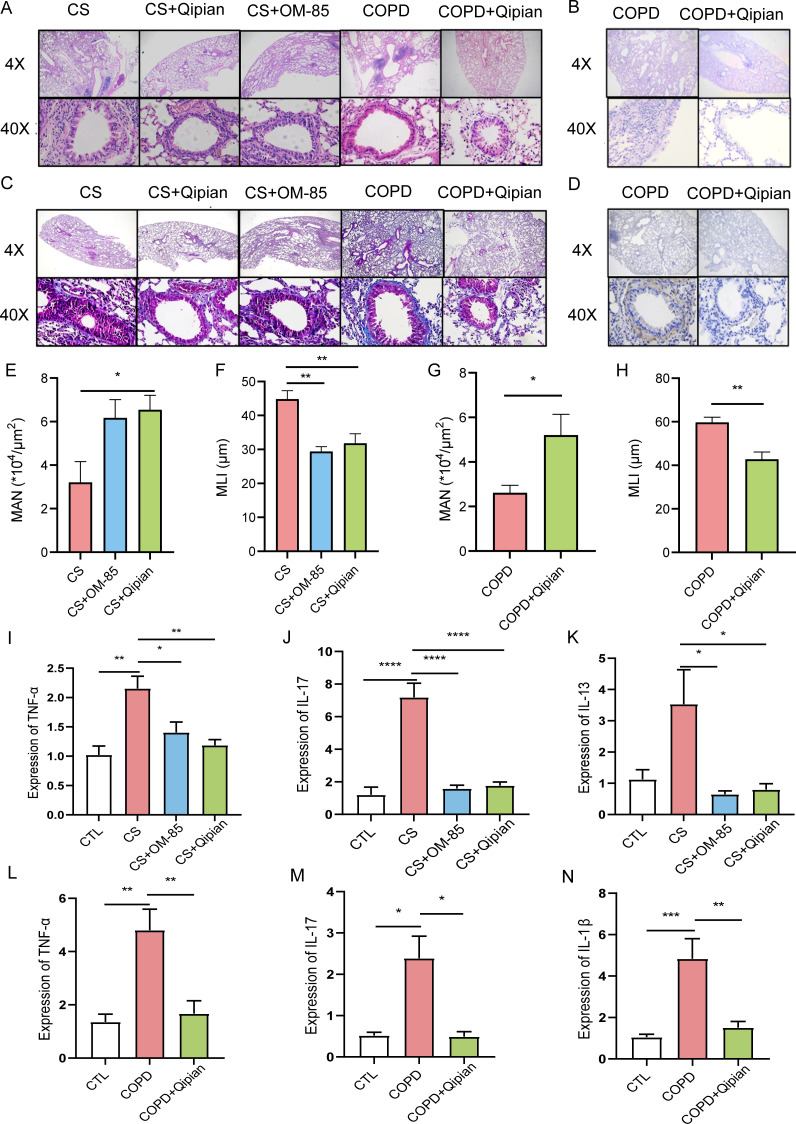
Pathological changes and expression of inflammatory cytokines in lung tissues. (**A**) HE staining. (**B**) PAS staining. (**C**) MT staining. (**D**) α-SMA immunohistochemistry. (**E, G**) Mean alveolar number. (**F, H**) Mean linear intercept. (**I–K**) Expression levels of TNF-α, IL-17, and IL-13 in the lung tissues in the model of prophylactic administration. (**L–N**) Expression levels of TNF-α, IL-17, and IL-13 in the lung tissues in the model of therapeutic administration. Data were expressed as mean ± standard error. *P < 0.05, **P < 0.01, ***P < 0.001, and ****P < 0.0001.

CS-exposed mice showed the disordered arrangement of airway epithelial cells, serious inflammatory cell infiltration, damaged alveolar structure, thickening of peribronchial smooth muscle, and increased collagen (Fig. 4A through D). Mice in the CS+Qipian group and COPD+OM-85 group exhibited slightly attenuated airway remodeling compared to those in the CS group. The lung sections from the mice in both the CS group and the COPD group exhibited alveolar enlargement and alveolar septal rupture, which are typical features of pulmonary emphysema. Conversely, lung sections of the CS+OM-85 group and CS+Qipian group showed a regular arrangement of alveoli and thin alveolar septa. Qipian and OM-85 treatment increased MAN and decreased MLI, indicating that Qipian could alleviate emphysema in the lungs of CS-exposed mice (Fig. 4E through H). Moreover, CS exposure increased the expression levels of tumour necrosis factor-alpha (TNF-α), interleukin-17 (IL-17), interleukin-13 (IL-13), and interleukin-1β (IL-1β) (Fig. 4I through N). However, Qipian and OM-85 were found to attenuate the CS-induced increase in the expression of inflammatory cytokines (Fig. 4I through N). Taken together, Qipian treatment could reduce inflammation and alleviate lung injury, and the effect was equal to that of OM-85. These effects were observed in both prophylactic and therapeutic models.

### Gut microbiota

**Fig 5:**
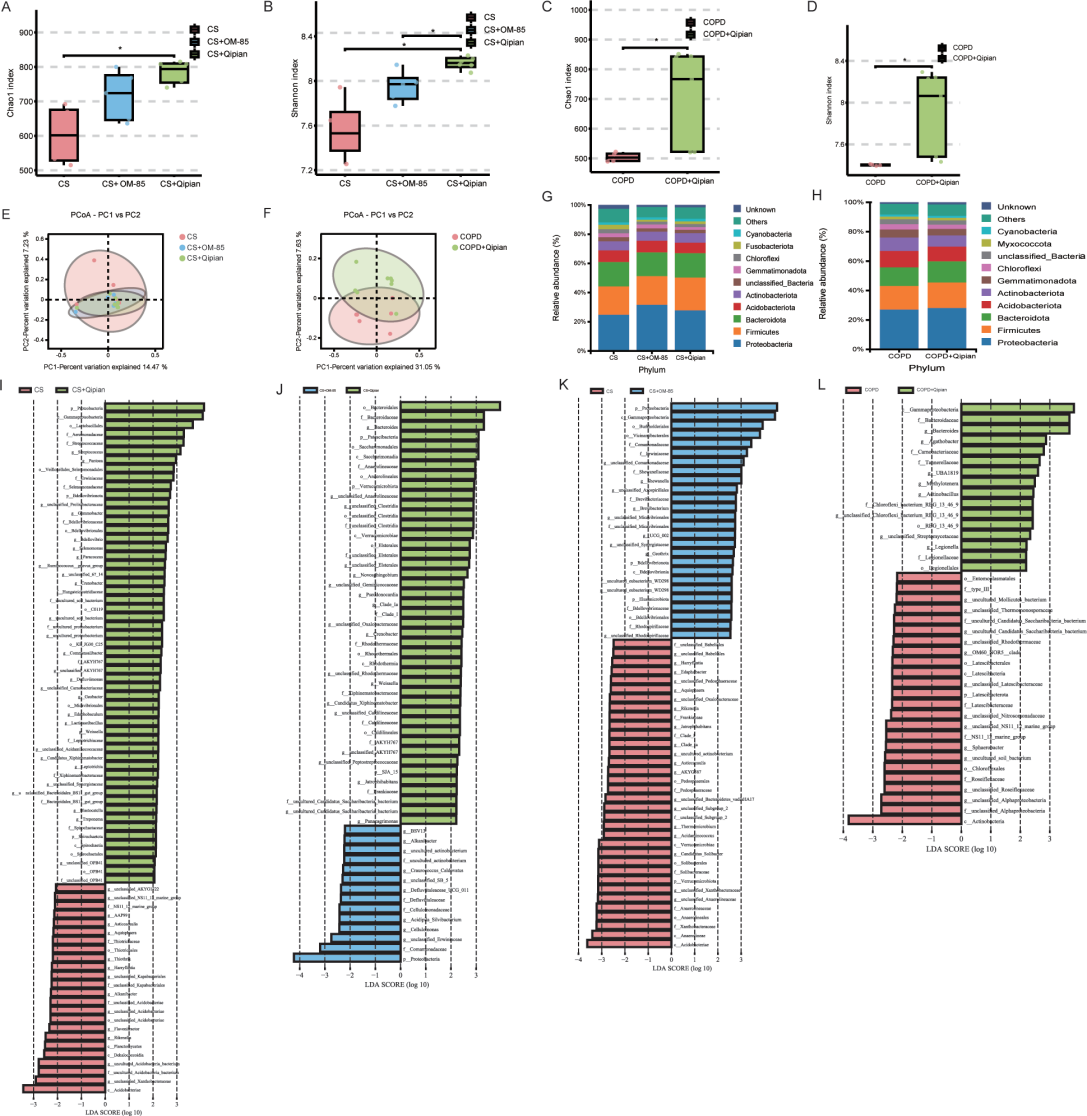
Gut microbiota analysis. (**A, B**) Chao1 and Shannon indexes in the model of prophylactic administration. (**C, D**) Chao1 and Shannon indexes in the model of therapeutic administration. (**E, F**) Principal coordinates analysis of gut microbiota in the models of prophylactic and therapeutic administration. (**G, H**) Histogram of gut microbiota distribution at the phylum level in the models of prophylactic and therapeutic administration. (**I–K**) LEfSe analysis in the model of prophylactic administration. (**L**) LEfSe analysis in the model of therapeutic administration. *P < 0.05.

The alpha and beta diversities were measured to assess differences in the microbiota community. In the model of prophylactic administration, the Chao1 index and Shannon index showed significant increases in the CS+Qipian group (*P* < 0.05) as well as an increasing tendency in the CS+OM-85 group (Fig. 5A and B). Similarly, in the model of therapeutic administration, Qipian treatment significantly increased the Chao1 index and Shannon index (*P* < 0.05) (Fig. 5C and D). PCoA revealed that the gut microbiota composition altered in both models of prophylactic and therapeutic administration (Fig. 5E and F). In the model of prophylactic administration, the CS+Qipian group and the CS+OM-85 group had similar gut microbiota composition, which differed significantly from that of the CS group. The comparison of bacterial abundance at the phylum level revealed increases in the relative abundance of Firmicutes and Proteobacteria in both the CS+Qipian group and the CS+OM-85 group (Fig. 5G). In the model of therapeutic administration, the relative abundance of Firmicutes and Bacteroidetes increased in the COPD+Qipian group (Fig. 5H). LEfSe analysis revealed significant differences between the CS group and the CS+Qipian group in Proteobacteria, *Lactobacillales*, *Bacteroidales*,* Lacticaseibacillus*, and so on (Fig. 5I through K). Furthermore, there were significant differences between the COPD group and the COPD+Qipian group in *Bacteroidaceae*, *Bacteroidales*, *Alphaproteobacteria*, and so on (Fig. 5L). Both prophylactic and therapeutic administration of Qipian resulted in changes in the relative abundance of gut bacteria, indicating that Qipian altered the composition of gut microbiota, increased the number of beneficial bacteria, and increased the richness and evenness of gut microbiota.

### Lung microbiota

**Fig 6:**
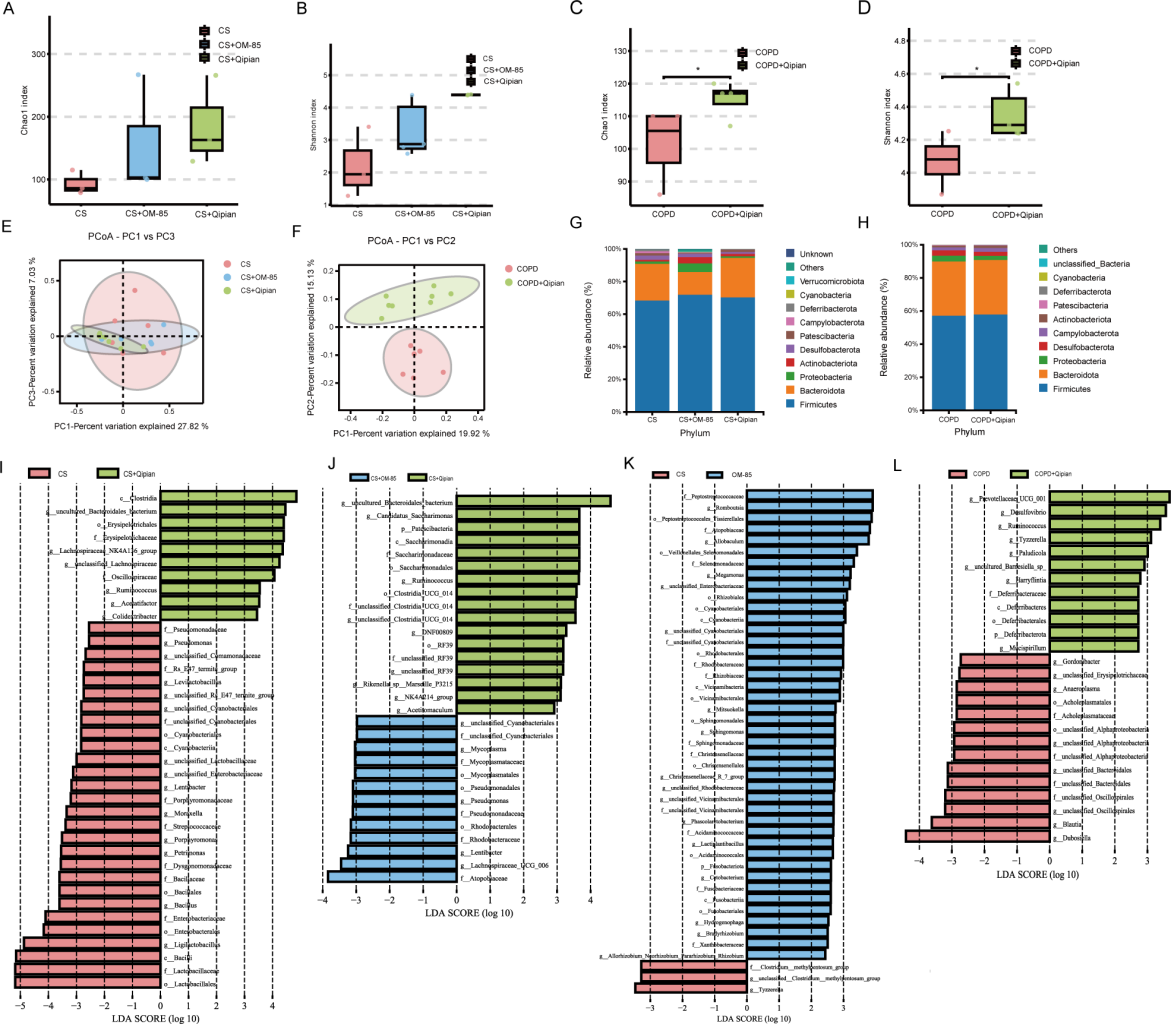
Lung microbiota analysis. (**A, B**) Chao1 and Shannon indexes in the model of prophylactic administration. (**C, D**) Chao1 and Shannon indexes in the model of therapeutic administration. (**E, F**) Principal coordinates analysis of lung microbiota in the models of prophylactic and therapeutic administration. (**G, H**) Histogram of airway microbiota distribution at the phylum level in the models of prophylactic and therapeutic administration. (**I–K**) LEfSe analysis in the model of prophylactic administration. (**L**) LEfSe analysis in the model of therapeutic administration. *P < 0.05.

Similar to the gut microbiota, the Chao1 and Shannon indexes for lung microbiota increased in both the CS+OM-85 and CS+Qipian groups in the model of prophylactic administration (Fig. 6A and B) and significantly increased in the COPD+Qipian group in the model of therapeutic administration (*P* < 0.01) (Fig. 6C and D). PCoA revealed that the composition of lung microbiota altered in both models of prophylactic and therapeutic administration (Fig. 6E and F). CS+Qipian and CS+OM-85 groups had similar microbiota composition. The comparison of bacterial abundance at the phylum level showed an increase in the abundance of Firmicutes and Bacteroidetes after Qipian treatment in both models of prophylactic and therapeutic administration (Fig. 6G and H). LEfSe analysis revealed that there were significant differences between the CS+Qipian group and the CS group in *Bacteroidales*, *Lachnospiraceae*, *Lactobacillales*, *Ligilactobacillus*, and so on (Fig. 6I through K). There were significant differences between COPD+Qipian and COPD groups in *Prevotellaceae*, *Desulfovibrio*, *Bacteroidales*, and so on (Fig. 6L). Both prophylactic and therapeutic administration of Qipian resulted in changes in the relative abundance of various respiratory bacteria. This suggested that the administration of Qipian could alter the composition of the lung microbiota, increase the number of beneficial bacteria, and increase the richness and evenness of the lung microbiota.

### Correlations of microbiota and inflammatory cytokines

**Fig 7:**
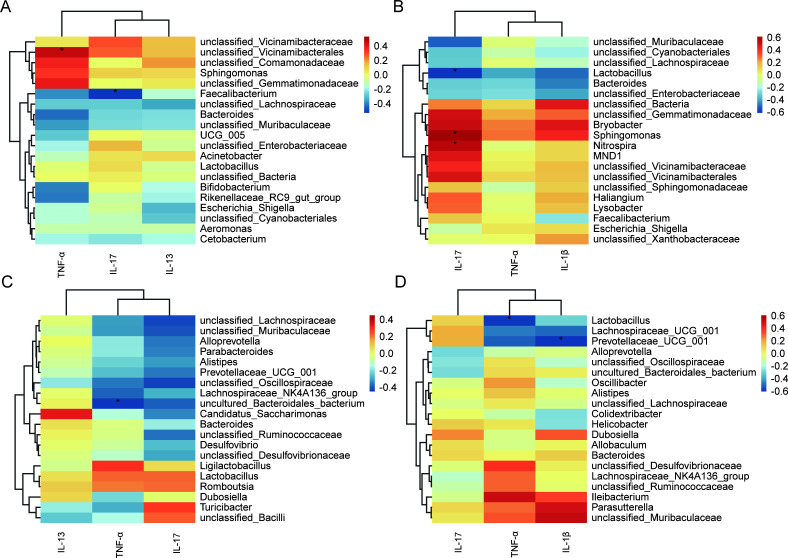
Correlations of microbiota and inflammatory cytokines. (**A**) The correlation between gut microbiota and inflammatory cytokines in lung tissues of mice in the prophylactic administration model. (**B**) The correlation between gut microbiota and inflammatory cytokines in lung tissues of mice in the therapeutic administration model. (**C**) The correlation between lung microbiota and inflammatory cytokines in lung tissues of mice in the prophylactic administration model. (**D**) The correlation between lung microbiota and inflammatory cytokines in lung tissues of mice in the therapeutic administration model.

In the model of prophylactic administration, Spearman correlation analysis showed that TNF-α expression was significantly positively correlated with *Vicinamibacterales* in gut microbiota, while IL-17 expression was significantly positively correlated with *Faecalibacterium* in gut microbiota (Fig. 7A); there was a significant negative correlation between TNF-α expression and *Bacteroides* in lung microbiota (Fig. 7B). In the model of therapeutic administration, the expression of IL-17 was significantly negatively correlated with *Lactobacillus* in the lung microbiota and significantly positively correlated with *Sphingomonas* and *Nitrospira* in the gut microbiota (Fig. 7C); the expression of TNF-α was negatively correlated with *Lactobacillus* in the lung microbiota (Fig. 7D); and the expression of IL-1β was negatively correlated with *Prevotellaceae* in the lung microbiota (Fig. 7D). These above findings suggested that microbiota were associated with inflammatory cytokines. Qipian might regulate the gut microbiota, increase the number of dominant gut microbiota, improve intestinal health, regulate the lung microecology through the gut-lung axis, and effectively reduce airway inflammation.

### TLR4/NF-κB pathway

**Fig 8:**
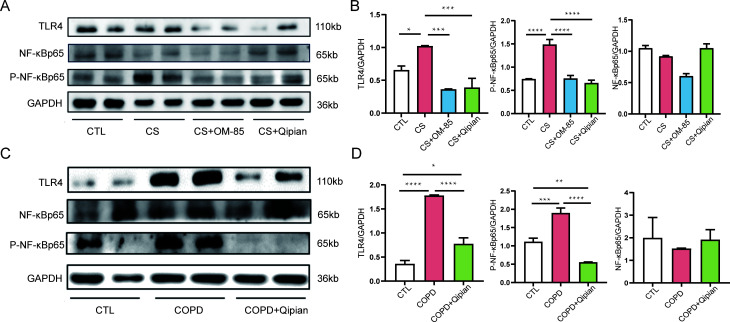
Effects of Qipian on the TLR4/NF-κB signaling pathway. (**A, B**) Western blot was performed to detect the expression of TLR4, p-NFkBp65, and NF-κBp65 in the model of prophylactic administration. (**C, D**) Western blot was performed to detect the expression of TLR4, p-NF-κBp65, and NF-κBp65 in the model of therapeutic administration. *P < 0.05, **P < 0.01, ***P < 0.001, and ****P < 0.0001.

Qipian treatment could reduce airway inflammation and alleviate lung injury by regulating the lung microbiota. The TLR4/NF-κB pathway might be involved in the underlying molecular mechanism. Compared to the control group, CS exposure upregulated the expression of intermediates in the TLR4/NF-κB pathway and NF-κB phosphorylation. Qipian and OM-85 significantly decreased TLR4 expression and prevented NF-κB phosphorylation (Fig. 8).

## DISCUSSION

COPD is characterized by marked emphysema and reduced lung function. In this study, Qipian treatment alleviated airway inflammation and remodeling, as well as improved lung function, possibly by regulating lung and gut microbiota.

Previous studies have proven that PBLs can alleviate symptoms such as cough and dyspnea in COPD patients, reduce the number of acute exacerbations, and decrease the number of subjects who require antibiotic treatment during acute exacerbations (20). OM-85 is an endotoxin-low lyophilized extract containing a cocktail of TLR ligands derived from eight major respiratory tract bacterial pathogens (*Haemophilus influenzae*, *Streptococcus pneumoniae*, *Streptococcus pyogenes*, *Streptococcus viridians*, *Klebsiella pneumoniae*, *Klebsiella ozaenae*, *Staphylococcus aureus*, and *Neisseria catarrhalis*) (21). Previous studies found that OM-85 reduced inflammation in pulmonary blood vessels and airways and reduced exacerbations caused by respiratory infections (22–25). In our study, Qipian treatment showed similar effects to OM-85 treatment. In summary, both Qipian and commonly used OM-85 in clinical practice can improve lung function by reducing airway inflammation and remodeling, thereby preventing and treating COPD.

Growing evidence has indicated that microbiota in the respiratory tract differs between healthy subjects and COPD patients, shifts in composition during COPD exacerbations, and varies among exacerbation subtypes, all suggesting a close association between the lung microbiota and COPD pathophysiology (26–30). Bowerman et al. (31) found that the fecal microbiome and metabolome of COPD patients were distinct from those of healthy individuals. The richness of gut microbiota in COPD patients was significantly lower than that in healthy individuals. Chiu et al. (32) analyzed the gut microbiota of 60 patients with COPD of varying severity and found that the abundance of *Bacteroides* was negatively correlated with eosinophil count and positively correlated with FEV1 and FVC, suggesting that the abundance of gut microbiota was associated with COPD severity. Our study also found that CS exposure altered microbiota composition, decreased microbiota richness and diversity, as well as significantly reduced the abundance of Bacteroidetes in both lung microbiota and gut microbiota in mice. A previous study suggested that treatment with PBLs acting on gut microbiota could promote heterologous immunomodulation useful in the prevention of recurrent respiratory tract infections and of wheezing inception and persistence (24). A clinical study suggested that the use of OM-85 might play a role in microbiota rearrangement (16). Consistent with other studies of PBLs, our study found that Qipian treatment altered microbiota composition, increased microbiota richness and diversity, and increased the abundance of Bacteroidetes and *Lactobacillus* in both gut and lung microbiota. Interestingly, prior studies have found that both *Bacteroides* and *Lactobacillus* are beneficial bacteria (16, 33). Belonging to the Bacteroidetes phylum, *Bacteroides* is a gram-negative, non-spore-forming, obligate anaerobic bacteria normally found in the human intestines, mouth, upper respiratory tract, and genital tract. *Bacteroides* expresses polysaccharide A, which can induce regulatory T-cell growth and cytokine expression that are protective against inflammation (16). Therefore, Qipian treatment could regulate the gut and lung microbiota and increase the beneficial bacteria both in the gut and lung.

A previous study showed that, in COPD patients, the lung microbiota was significantly associated with pro-inflammatory markers in sputum, especially IL-8 (28). Sputum IL-8 was significantly correlated with both alpha and beta diversities of the lung microbiota in COPD patients (28). Our study found that *Bacteroides* and *Lactobacillus* were negatively correlated with some inflammatory cytokines, and *Vicinamibacterales*, *Faecalibacterium*, *Sphingomonas*, and *Nitrospira* were positively correlated with these cytokines in lung tissues, indicating that regulating gut microbiota can alleviate lung inflammation. Qipian treatment might improve the imbalance of gut microbiota and maintain airway microecological homeostasis through the gut-lung axis, thus alleviating lung inflammation and exerting immunomodulatory effects.

Gut microbiota imbalance might regulate the TLR4/NF-κB pathway in the pulmonary immune system, which activates oxidative stress in the lung, mediates lung injury via regulating the intestinal barrier, and increases the expression of inflammatory factors, including TNF-α, IL-1β, and IL-6 (17). A prior study has shown that, as a vital pattern recognition receptor, TLR4 can identify pathogenic bacteria that have translocated into the blood circulation, thereby initiating downstream signal transduction pathways and ultimately activating the NF-κB pathway (17). Di Stefano et al. (18) found that bronchial inflammation and bacterial load in stable COPD were associated with TLR4 overexpression. In a mouse model of LPS-induced acute lung injury, gut microbiota imbalance promoted the expression of TLR4 and the phosphorylation of NF-κB, as well as the expression of IL-6 and TNF-α, while fecal microbiota transplantation reduced TLR4 expression and suppressed NF-κB phosphorylation (17). Therefore, regulating gut microbiota reduces the release of inflammatory cytokines in the lungs. In addition, in the diesel exhaust particles-exposed mice model, the expansion of *Proteobacteria* in the airway may contribute to the activation of TLR4, promote the release of inflammatory cytokines, and form a vicious cycle of inflammation and microbiota imbalance (34). Previous studies have reported that polyphyllin B regulates gut microbiota and affects the TLR4/NF-κB pathway, thereby improving lung function as well as reducing airway damage, inflammation, and remodeling (35, 36). Our study also suggested that Qipian could suppress the TLR4/NF-κB pathway to exert prophylactic and therapeutic effects on COPD patients.

The regulation of pulmonary immunity and inflammatory response by gut microbiota also relies on some metabolites such as short-chain fatty acids (SCFAs). SCFA is a key molecule involved in the process in which gut microbiota regulates COPD through the gut-lung axis (37). However, this study did not detect the metabolome, so the impact of Qipian on the metabolites cannot be determined. Moreover, the study is conducted solely in a murine model, and while the results are promising, they may not directly translate to human COPD due to significant differences between human and mouse microbiota. The small number of animals per group could also limit the statistical power of the study. The clinical validation will be included in our future studies.

Anyway, this study had some strengths. (i) Innovative focus on the gut-lung axis: the study delves into the novel concept of the gut-lung axis and its role in COPD. This focus is timely and relevant, as recent studies have begun to highlight the bidirectional interactions between gut and lung microbiota and their implications for inflammatory lung diseases like COPD. (ii) Therapeutic and prophylactic models: the use of both prophylactic and therapeutic models provides a comprehensive view of Qipian’s potential effects. This dual approach strengthens the applicability of the findings in both prevention and treatment contexts. (iii) Microbiota analysis: the study’s microbiota analysis using 16S rRNA sequencing adds depth to the mechanistic understanding of how Qipian may influence disease progression through the modulation of both gut and lung microbiota. (iv) Multifaceted approach: the combination of techniques like lung function tests, histological analyses, Western blot for TLR4/NF-κB, and correlation analyses between microbiota and inflammatory cytokines enhances the study’s multidisciplinary nature, supporting more robust conclusions.

In summary, Qipian has both preventive and therapeutic effects on COPD. The mechanism may be that it regulates the lung microecology through the gut-lung axis and inhibits the activation of the TLR4/NF-κB pathway, thus reducing airway inflammation, improving lung function, and reducing irreversible lung injury. Our results provide a theoretical basis for the clinical application of Qipian in the prevention and treatment of COPD.

## Data Availability

The datasets used and/or analyzed during the current study are available from the corresponding author on reasonable request. The datasets generated and/or analyzed during the current study are available in the Sequence Read Archive (SRA) database, accession number: PRJNA1097331.

## SUPPLEMENTAL MATERIAL

Table S1 spectrum.01969-24-s0001.docx Primer sequences used for quantitative RT-PCR.

ASM does not own the copyrights to Supplemental Material that may be linked to, or accessed through, an article. The authors have granted ASM a non-exclusive, world-wide license to publish the Supplemental Material files. Please contact the corresponding author directly for reuse.
