# Supplementary material for: Therapeutic and prophylactic effects of Qipian on COPD in mice: the role of lung and gut microbiota
Source: Microbiol Spectr. 2025 Jul 14;13(8):e01969-24. doi: 10.1128/spectrum.01969-24 (PMC12327850; doi:10.1128/spectrum.01969-24)
Supplement: Table S1 — Primer sequences used for quantitative RT-PCR. [file spectrum.01969-24-s0001.docx]

**Supplementary Table 1** Primer sequences used for quantitative RT-PCR

| Gene | Forward primer (5′ to 3′) | Reverse primer (5′ to 3′) |
| --- | --- | --- |
| TNF-α | ACTCCAGGCGGTGCCTATGT | GTGAGGGTCTGGGCCATAGAA |
| IL-1β | AGTGTGGATCCCAAGCAATACCCA | TGTCCTGACCACTGTTGTTTCCCA |
| IL-13 | CTCTTGCTTGCCTTGGTGGTCTC | GGGAGTCTGGTCTTGTGTGATGTTG |
| IL-17 | TCAGCGTGTCCAAACACTGAG | CGCCAAGGGAGTTAAAGACTT |
| GAPDH | CATCACTGCCACCCAGAAGACTG | ATGCCAGTGAGCTTCCCGTTCAG |
